# The Emergence of Regional Immigrant Concentrations in USA and Australia: A Spatial Relatedness Approach

**DOI:** 10.1371/journal.pone.0126793

**Published:** 2015-05-12

**Authors:** Josef Novotny, Jiri Hasman

**Affiliations:** Department of Social Geography and Regional Development, Faculty of Science, Charles University in Prague, Prague, Czech Republic; Peking UIniversity, CHINA

## Abstract

This paper examines the patterns of the US and Australian immigration geography and the process of regional population diversification and the emergence of new immigrant concentrations at the regional level. It presents a new approach in the context of human migration studies, focusing on spatial relatedness between individual foreign-born groups as revealed from the analysis of their joint spatial concentrations. The approach employs a simple assumption that the more frequently the members of two population groups concentrate in the same locations the higher is the probability that these two groups can be related. Based on detailed data on the spatial distribution of foreign-born groups in US counties (2000–2010) and Australian postal areas (2006–2011) we firstly quantify the spatial relatedness between all pairs of foreign-born groups and model the aggregate patterns of US and Australian immigration systems conceptualized as the undirected networks of foreign-born groups linked by their spatial relatedness. Secondly, adopting a more dynamic perspective, we assume that immigrant groups with higher spatial relatedness to those groups already concentrated in a region are also more likely to settle in this region in future. As the ultimate goal of the paper, we examine the power of spatial relatedness measures in projecting the emergence of new immigrant concentrations in the US and Australian regions. The results corroborate that the spatial relatedness measures can serve as useful instruments in the analysis of the patterns of population structure and prediction of regional population change. More generally, this paper demonstrates that information contained in spatial patterns (relatedness in space) of population composition has yet to be fully utilized in population forecasting.

## Introduction

How do the distinct spatial choices of particular immigrant groups shape regional population change? Understanding this is high on the public policy agenda, in particular in countries with a tradition of immigration, such as USA and Australia, where immigration represents a major driver of population dynamics. This is even more evident at the regional level, which is also subject to regionally specific demographic and economic trends, and internal migrations of the native population. In many regions immigrants have become an indispensable part of labour markets, supplementing and sometimes substituting the native labour force. Regions with large inflows of immigrants have to deal with diverse challenges and consequences of immigration, including the effects on infrastructure capacity, availability of services, housing costs, employment levels, impacts on local wage equilibria and crime rates, among many others [1, 2, 3]. Despite the importance for population change and general population projections, immigration and the ethnic composition of the regional population is difficult to predict [4, 5, 6, 7, 8, 9, 10, 11].

This paper seeks to contribute to an understanding of the patterns of immigration and the process of regional population diversification in terms of the emergence of new regional concentrations of immigrant groups. Inspired by methodology used recently in very different contexts [12, 13, 14], our approach capitalizes on the concept of spatial relatedness as inferred from the analysis of spatial distribution of foreign-born population groups. The spatial relatedness is understood as a degree of co-occurrence of the members of two population groups in the same regions. Here it is analysed using a pair-wise proximity measure which quantifies the extent of joint concentrations of two population groups in regions. Essentially, the spatial relatedness approach is based on a simple assumption that the degree of co-occurrence (spatial co-concentration) increases the probability that the two groups can be related.

Although diverse, the literature on the spatial distribution of foreigners mostly focuses on between-region comparisons of population compositions, either by comparing the spatial patterns of immigration through maps or by quantitative comparisons of immigrant counts between some spatial units [1, 15, 16, 17, 18, 19, 20, 21, 22, 23]. Unlike the traditional literature, here we compare spatial patterns of individual immigrant groups themselves. Based on detailed data on the spatial distribution of foreign-born groups in 3,143 US counties and 2,513 Australian postal areas, we quantify and examine the pair-wise spatial relatedness between individual groups as revealed from the analysis of their joint concentrations in these spatial units. Using the pair-wise spatial relatedness figures we model the aggregate structure of migration systems in USA and Australia conceptualized as the undirected networks of foreign-born groups linked by their spatial relatedness. We then quantify the spatial relatedness of a given group to the pool of those groups already concentrated in a region. We assume that the current population composition of a region affects its future population structure, in that the emergence of new immigrant concentrations is more likely for groups with higher relatedness with population groups already concentrated in the region. The ultimate goal of this study is to examine the power of spatial relatedness measures in predicting changes in the population composition of regions in USA and Australia.

Although examining relatively short time spans (2000–2010 and 2006–2011 for USA and Australia, respectively), the past recent years are interesting for the proposed exercise because both of the countries have witnessed significant increase of foreign-born population (by 24% in USA and 20% in Australia) accompanied by important changes in their geography of immigration. The new developments such as declining (relative) importance of gateway cities and spatial deconcentration of many foreign-born groups interrelated with the emergence of new places of immigration [2, 15, 19, 20, 22, 24, 25, 26, 27, 28, 29] have made the projections of regional population change even more challenging, while somewhat devaluating common approaches based on extrapolations of past trends. The spatial relatedness approach presented here can help make these projections more precise and anticipate some of the above mentioned challenges related to immigration. Therefore, the general goal of this article is to demonstrate that information derived from the spatial patterns of population composition has yet to be fully utilized as a valuable source of inference for population forecasting.

The rest of this article has the following structure: The next section briefly overviews main factors determining spatial choices of immigrants and spatial relatedness between immigrant groups. This is followed by the description of methods and data used in our analysis. The results section firstly presents the statistical distribution of spatial relatedness observations and compares the sets of these observations obtained for the US and Australian data sets. Secondly, it provides the network representations showing the aggregate structure of the US and Australian immigration geography. The main part of the results section then provides several exercises examining the power of spatial relatedness measures in predicting the emergence of regional concentrations of foreign-born groups. The article closes with some concluding remarks in the final section.

## Factors behind the spatial relatedness of foreign-born groups

The basic assumption behind the approach applied here is that the degree of spatial relatedness between immigrant groups signifying the degree of similarity in their destination choices can mirror various other aspects of their relatedness based on, for example, common geographical origin, cultural and historical similarities or similarities in various political and economical characteristics. The voluminous literature on the determinants of spatial distribution of immigrants (for an overview, see [7, 30, 31]) provides us with several arguments in support of the assumed parallels between spatial relatedness and other forms of relatedness, only a few of which we can briefly touch upon here. Cultural proximity is often attributed a key role, though economic, geographical, and political factors also matter. Perhaps the most common underlying mechanism refers to the power of migration social networks in determining spatial concentrations of specific immigrant groups [32]. It is also thought that migration networks can have somewhat differential impacts on destination choices depending on migrants’ skills [3], race [15, 33], refugee status [28, 34], or language. A lack of knowledge of the host country’s language can strengthen the power of migration networks. In some cases, this may even lead to economically sub-optimal destination choices [35]. As documented for Australia, for instance, migrants without local language proficiency tend to be over-represented in the largest cities, where they have a greater opportunity of finding compatriots [28, 36, 37]. By contrast, the evidence from Portugal suggest that migrants who speak the local language prefer cities with more employment opportunities in services where language knowledge is important, while migrants without local language proficiency tend to opt for work in agriculture in more peripheral regions [18]. Although obviously context-dependent, such examples show that language knowledge might be an important marker of cultural relatedness that structures immigrants’ choice of destination.

Economic models typically regard immigrants as rational agents who utilize their capabilities (knowledge, skills and experiences), in order to maximize the benefits and minimize the costs of migration, what also determines their spatial behaviour [19, 38]. Implicitly, it is assumed that migrants of similar origin would often have similar capabilities and deal with similar constraints and, therefore, would reveal similar destination choices [39, 40]. The focus on matching the economic characteristics of migrants with their source and target destinations is, nevertheless, in contradiction to the simplistic neoclassical theories that argue that migrants would, regardless of their skills, automatically prefer the regions with the highest wages (or lowest unemployment rates) until regional differences diminish.

Because of the additional cost of migrating to more remote areas, spatial distance between place of origin and target destination can also be a potentially influential factor in terms of the costs of moving both between countries [41] and within them [42]. As such, immigrants from the same source country can be expected to head to similar (easily accessible) destinations, though this would obviously also depend on other factors, such as migrants’ skills and economic resources [43].

There have also been attempts to place administrative restrictions on the movement of immigrants with lower human capital, and these restrictions are often tightly interwoven with the migrants’ source country and other markers [39, 40, 44]. Rather than the opposite, such selective administrative measures contribute to the emergence of spatial concentrations of specific foreign-born groups.

Finally, the historical context of immigration also has an important impact on spatial patterns. In conjunction with other factors, the length of stay shapes the degree of spatial concentration and spatial assimilation of particular immigrant groups. Regarding the two countries in this study, it is well known that immigration can be divided into several successive historical waves. These waves have been distinguished on the basis of changes in the political, economic, social and demographic context both in the source and host countries, and signified by the different composition of incoming migrants, as well as by their different destination choices [28]. Through migration networks, immigrant settlements established in particular waves have attracted, and continue to attract, other migrants from the same or related groups.

## Methods

From several possible relatedness indices, we applied a pair-wise measure that quantifies the frequency of joint concentrations of two population groups (here defined by country of birth) in the same regions. The concentration of a population group (*i*) in a region (*r*) was expressed using the common localization quotient (*LQ*_*i*,*r*_) formally written as:

LQi,r=Fi,r∑iFi,r∑rFi,r∑i∑rFi,r (1)

where *F*_*i*,*r*_ denotes the size of the population group *i* in the region *r*. *In the following text we say that group i concentrates in region r if the condition of LQ*_*i*,*r*_ *> 1 is met*. *LQ*_*i*,*r*_ > 1 indicates that the given group is more prevalent in the population of region *r* than in the entire population of the country. The threshold of 1 was applied here because of its intuitively appealing interpretation. Moreover, we noted that that the consideration of other limiting values of *LQ*_*i*,*r*_ (we tested a few other reasonable values between 0.8 and 3.0) only marginally influence the results.

The size of the set of regions in which two population groups (*i*, *j*) established regional concentration (denoted as {*r*: *LQ*_*i*,*r*_ > 1} and {*r*: *LQ*_*j*,*r*_ > 1}) is the key input for measuring the extent of joint concentrations of the two population groups (*i*, *j*). We applied a combination of the Dice asymmetric coefficients, adjusted as in [13]. The first asymmetric Dice measure captures the probability that group *i* concentrates in region *r* conditional to the concentration of group *j* in the same region:

$$D_{\text{i}|\text{j}}^{1}=P\left(LQ_{i,r}>1|LQ_{j,r}>1\right)=\frac{\left|\left\{r:LQ_{i,r}>1\right\}\cap \left\{r:LQ_{j,r}>1\right\}\right|}{\left|\left\{r:LQ_{j,r}>1\right\}\right|}$$ (2)

where the numerator represents the size of the set of regions in which both groups *i* and *j* are concentrated and the denominator represents the size of the set of regions in which group *j* concentrated.

Analogously, the second Dice measure corresponds to the probability that group *j* concentrates in region *r* conditional to the concentration of group *i* in the same region:

$$D_{\text{j}|\text{i}}^{2}=P\left(LQ_{j,r}>1|LQ_{i,r}>1\right)=\frac{\left|\left\{r:LQ_{i,r}>1\right\}\cap \left\{r:LQ_{j,r}>1\right\}\right|}{\left|\left\{r:LQ_{i,r}>1\right\}\right|}$$ (3)

In order to obtain a symmetric measure that would keep an intuitively appealing interpretation as the conditional probability of joint concentration, we considered the smaller of the two asymmetric Dice indices described above. Note that if a group *i* concentrates in solely one region then the second Dice measure always attains its maximal value of 1 with respect to its relatedness to all those groups *j* that are also concentrated in the same region irrespective of the number of concentrations of group *j* in other regions. Since we wish to capture the degree of similarity in the destination choices of the immigrant groups, it makes sense to consider the smaller of the asymmetric Dice indices so the pair-wise relatedness measure used for our analysis was denoted as:

$$D_{i,j}=\text{min}\left(D_{i|j}^{1};D_{j|i}^{2}\right)$$ (4)

This ranges between 0 and 1; the minimum value is attained when the two groups concentrate exclusively in different regions and the maximum value is reached when these two population groups concentrate exclusively in the same regions. The actual value of *D*_*i*,*j*_ can be interpreted as the probability that one of these groups concentrates in a region where another is concentrated.

To examine our central goal, we further considered a measure of the density around a population group (*i*) in a region (*r*) in terms of the average relatedness between this group and all other groups already concentrated in the region. The density measure was formalized analogously as in [12]:

wi,r=∑kxk,rDi,k∑kDi,k (5)

where *x*_*k*,*r*_ is 1 if group *k* concentrates in region *r*, and 0 if otherwise. We employed this measure to test whether the spatial relatedness can be used to predict regional population diversification in terms of the emergence of settlement concentrations of new immigrant groups in a region.

## Data

For each of the countries we used data from the year of the most recent census, and data from the year of the census prior to that. This enabled us to examine the change that occurred during the period between the censuses. We applied data disaggregated by population groups, as defined by the country of birth and by spatial units, namely the county-level data in USA (3,143 counties) and data at the level of postal areas in Australia (2,513 postal areas). The source of US data was the US Census Bureau. We obtained the US data through a publicly accessible application American FactFinder (http://factfinder.census.gov). Through this interface, we obtained data from the 2006–2010 American Community Survey (combining information from the Population Estimates Program 2006–2009 and the US Census 2010) and older data from the 2000 US Census. For Australia, we employed data from the 2011 and 2006 Australian Censuses of population obtained through a publicly accessible interface (https://www.censusdata.abs.gov.au/webapi) operated by the Australian Bureau of Statistics. As the Australian small-number data cells (a cell corresponds to the size of a certain foreign-born group in a spatial unit) were randomly adjusted by the Australian Bureau of Statistics because of privacy protect policy, we considered only those foreign-born groups with the aggregate size of more than 500 individuals.

Although we covered relatively short periods, we already mentioned that the foreign-born population increased significantly in both countries with considerably faster growth rates among immigrants from developing countries (especially countries in Asia and Africa). There were some minor changes in the number of spatial units in both countries over the considered periods, so we had adjust the data appropriately in order to make the data sets comparable wherever direct comparisons were undertaken. The basic descriptive characteristics of the data sets are presented in Table 1.

**Table 1:** Basic descriptive characteristics of the data.

	USA	Australia
Foreign-born population total	38,674,773	5,254,493
Share of foreign-born in total population	0.127	0.245
Nm. of foreign-born population groups	133 (96)	153 (137)
Nm. of spatial units; counties in USA and postal areas in Australia	3,143 (3,116)	2,513 (2,407)
Average share of foreign-born population in regional population	0.044	0.172
Std. deviation of average share of foreign-born population in regions	0.056	0.121
Average region population size	96,712	8,539
Standard deviation of average region population size	308,504	11,443

Note: The figures in parentheses refer to adjusted data sets compatible between different years.

Based on the data sources described above, for each of the countries we obtained two data matrices containing the population counts of particular foreign-born groups in individual spatial units at two points in time. For each of the matrices, we calculated the localisation quotients (*LQ*_*i*,*r*_) and assessed whether particular groups concentrate in particular regions or not. Based on these results we then calculated the measures of spatial relatedness (*D*_*i*,*j*_, *w*_*i*,*r*_) as explained above and proceeded to the further analysis presented in the Results section below.

## Results

### The patterns of spatial relatedness

**Fig 1:**
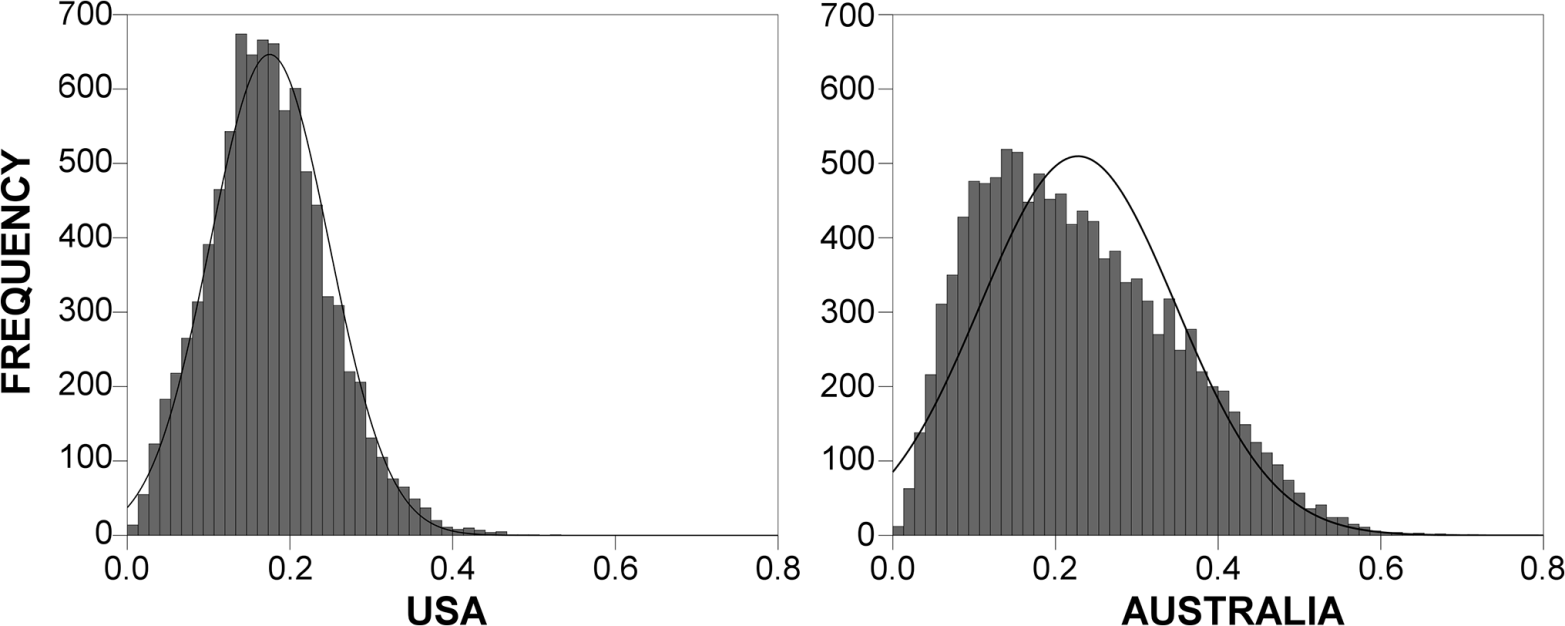
Distributions of spatial relatedness links *D*_*i*,*j*_.

Our first step was to calculate the matrices of pair-wise spatial relatedness indices (*D*_*i*,*j*_) between all possible pairs of immigrant groups. Fig 1 presents distributions of these results for the more recent years. The distribution for Australia is more right-skewed, having both a larger number of small values and more high-value observations on the right side. This can be attributed to more unequal population distribution across Australian regions with respect to both the distribution of the total and foreign-born population, in combination with generally higher shares of immigrants in the Australian population (Table 1). We noted that many of the small observations on the left side of the plots in Fig 1 may be regarded as noise associated with random co-occurrences of immigrants in regions. Thus, we were particularly interested in the right-hand side of the plots, with more significant *D*_*i*,*j*_ links. This is particularly true for the longer right-tails of the plots composed of the most significant *D*_*i*,*j*_ links, of which the 15 highest observations are listed in Table 2. Using the conditional probability interpretation of *D*_*i*,*j*_ measure as applied here, we can say that, for example, Malaysian and Singaporean immigrant groups in Australia have a 71.7% probability that one of these groups concentrates in a region where another is concentrated. An inspection of Table 2 confirms that the highest spatial relatedness observations can be found between groups from geographically and culturally proximate countries. At the same time, the composition of source countries listed in the table mirrors the known distinctions in the US and Australian immigration systems; this relates primarily to the prominence of geographically proximate source countries.

**Table 2:** The 15 most significant spatial relatedness links *D*_*i*,*j*_.

	USA			Australia		
1.	Jamaica	Trinidad and Tobago	0.524	Malaysia	Singapore	0.717
2.	China	Taiwan	0.496	Hong Kong	Korea, South	0.691
3.	West Indies	Trinidad and Tobago	0.487	Hong Kong	Indonesia	0.677
4.	Barbados	Jamaica	0.478	Hong Kong	Malaysia	0.668
5.	Jamaica	Colombia	0.463	Canada	USA	0.665
6.	India	Pakistan	0.462	Hong Kong	Taiwan	0.650
7.	Haiti	Jamaica	0.458	Cyprus	Greece	0.649
8.	Ecuador	Dominican Republic	0.457	Hong Kong	Iran	0.643
9.	Jamaica	West Indies	0.454	China	India	0.639
10.	Grenada	Guyana	0.452	China	Malaysia	0.630
11.	Jamaica	Guyana	0.443	Taiwan	Korea, South	0.627
12.	India	Egypt	0.442	Indonesia	Malaysia	0.624
13.	China	Hong Kong	0.441	Iran	Malaysia	0.621
14.	Korea, South	India	0.437	China	Hong Kong	0.615
15.	Italy	Poland	0.435	Hong Kong	Singapore	0.614

S1 and S2 Figs provide an illustrative outline of the US and Australian geography of immigration by depicting the aggregate patterns of spatial relatedness using network visualisations (because of size limitations it was not possible to include these visualisations in the main body of the present paper). Note that this type of the network representation of immigration patterns differs from recent attempts to examine (real) human migration networks [45, 46]. In the present paper, the networks are undirected and nodes represent countries of birth of individual population groups and the edges correspond to the spatial relatedness links (*D*_*i*,*j*_). To generate these networks we used a spring-embedded algorithm with consideration of weights linearly proportional to the values of *D*_*i*,*j*_. The nodes are coloured according to the respective world regions of individual source countries (see legend placed in the top left corner of the pictures) and their size is proportional to the square root of the population size of particular groups. Although all observations with *D*_*i*,*j*_ > 0.2 were considered for creating the networks, only edges with *D*_*i*,*j*_ above 0.30 and 0.45 for USA and Australia, respectively, are displayed in order to keep the network plots readable. Each of the networks in S1 and S2 Figs also contains the biggest node for native population that serves as the reference point indicating the degree of dissimilarity of particular foreign-born groups from the spatial distribution of native population.

Although a more detailed discussion of the network visualisations is beyond the scope of this paper, in general these graphs again show obvious parallels between the spatial relatedness and geographical and cultural relatedness of the individual source countries and their communities as they emerged in the networks. In both network graphs, the biggest nodes for native population are neighboured by the community of Western countries (and South Africa) followed by Central and Eastern European countries. Most of the other countries then occupy positions that are more distant from that of native population and they often form pairs or communities of geographically and culturally related ones. Especially the US network plot (S1 Fig) shows a clear clustering of the Latin American and Caribbean source countries. An exception is a unique position of Mexico, far the most populous non-native group with a special and generally relatively dispersed pattern of spatial distribution across US regions. An interesting example of a factor structuring the network graphs is the sequence of the waves of immigration. This can be illustrated by an inspection of the positions of typical source countries of individual waves of immigration to Australia (Table 3) in S2 Fig and particularly by the distance of their respective nodes to that pertaining to native population.

**Table 3:** Typical source countries for the waves of immigration to Australia.

Period	Source countries or regions
Turn of 1940s and 1950s	Eastern Europe
Early 1950s	The Netherlands, Germany
Late 1950s	Italy, Greece, Yugoslavia
1960s	Lebanon, Turkey
1970s-1990s	Asia (successively South-East, Eastern and Southern)
2000s	Africa

Note: Based on [28]. Note that the most traditional source countries such as the UK, Ireland, and New Zealand have for most of the time been important for Australian immigration.

**Fig 2:**
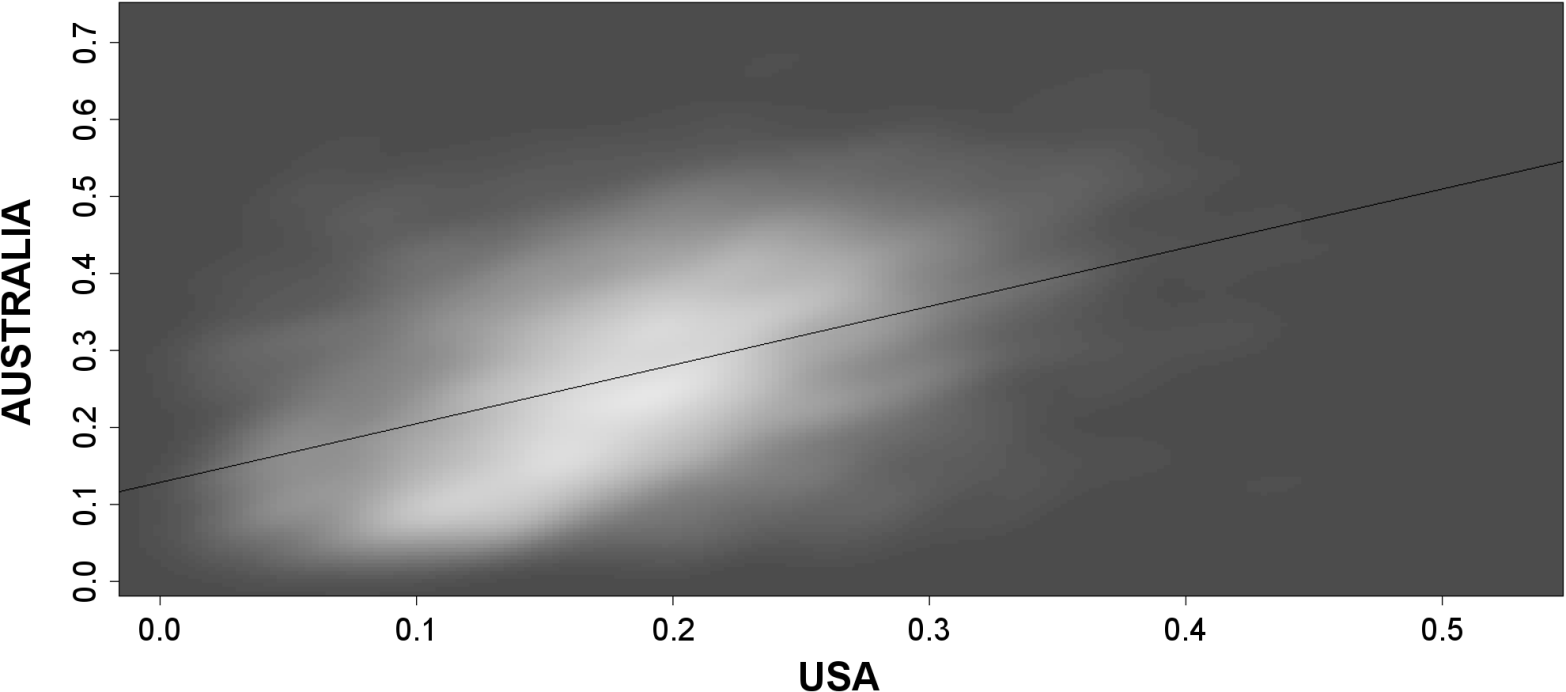
The relationship between *D*_*i*,*j*_ for USA and Australia: bivariate kernel density plot Note: The lighter colours, the higher density of *D*_*i*,*j*_ observations.

One intriguing question behind our analysis was whether there is a similarity in the spatial choices of particular immigrant groups in USA and Australia. Such a similarity would again suggest the relevance of the approach taken in this paper, in terms of the existence of factors structuring the spatial choices of particular immigrant groups independently of the specific context of a particular country. To explore this question we filtered the 5,560 *D*_*i*,*j*_ links between the 96 immigrant group that appear in the data from both USA and Australia. We found a statistically significant correlation of 0.454 between these two sets of *D*_*i*,*j*_ results. This can be considered a relatively close relationship, especially considering the noise in the data associated with lower values of *D*_*i*,*j*_ and random co-occurrences of immigrants. A more informative illustration of this relationship is provided in Fig 2, which uses a bivariate kernel density plot to depict these sets of *D*_*i*,*j*_ observations obtained for both countries.

**Fig 3:**
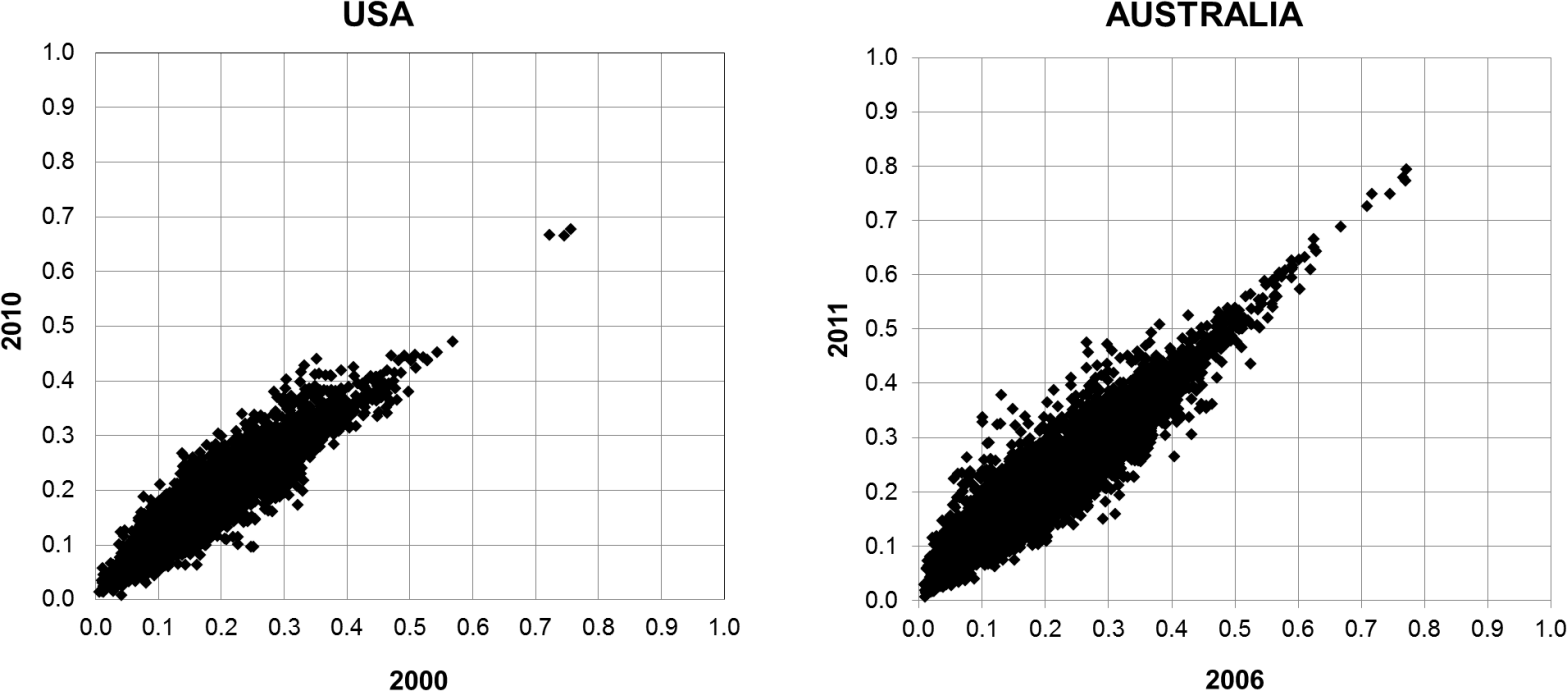
The time stability of the *D*_*i*,*j*_ observations Note: The plots display 4,560 pair-wise *D*_*i*,*j*_ observations between 96 immigrant groups in USA (left side) and 9,316 observations between 136 immigrant groups in Australia (right side).

Yet, an even more important question with respect to the approach presented in this paper was whether there is a time stability of *D*_*i*,*j*_ observations. Obviously, if the patterns of spatial relatedness substantially fluctuate in time, then the utility of our approach may be questioned. To test the stability of *D*_*i*,*j*_ observations we confronted the compatible sets of the observations for 20002010 and 2006–2011 for USA and Australia, respectively. These compatible sets corresponded to 4,560 pair-wise *D*_*i*,*j*_ observations between 96 immigrant groups in USA and to 9,316 observations between 136 immigrant groups in Australia. Importantly, for both countries we found strong relationships between the sets of observations (Fig 3) with the correlation coefficients of 0.913 and 0.942 for USA and Australia, respectively. These findings suggest a considerable time stability of the spatial relatedness measures and support their applicability for the present purposes.

### The spatial relatedness and emergence of new immigrant concentrations in regions

As follows from the methodology described above, immigrant group *i* can either be concentrated in region *r* (meaning that *LQ*_*j*,*r*_ > 1) or it can be un-concentrated in this region (*LQ*_*j*,*r*_ ≤ 1). We noted that un-concentrated groups are considerably more frequent, accounting for more than 80% of all observations in each data set used here. Tables 4 and 5, representing USA and Australia, respectively, explore the distributions of concentrated and un-concentrated groups according to different levels of density around group *i* in region *r* (*w*_*i*,*r*_ as defined above). In addition, these tables also examine the relationship between the density *w*_*i*,*r*_ and the emergence of new immigrant concentrations in regions. These results provide two important findings with respect to the central research question of this paper. First, it is shown that the density *w*_*i*,*r*_ is significantly positively associated with higher probability of concentration (the fourth columns of Tables 4 and 4). For example, the probability of concentration within the most frequent category of *w*_*i*,*r*_ < 0.1 corresponds to less than 5% for USA and to 2% for Australia. By contrast, for all of the groups with *w*_*i*,*r*_ > 0.4, this probability stands at 50% and 63% for USA and Australia, respectively. These results clearly show that the density (average spatial relatedness to the groups already concentrated in a given region) strongly predict regional concentrations of immigrant groups. Second, from a more dynamic perspective, the density *w*_*i*,*r*_ was also confirmed as a significant predictor of new immigrant concentrations in regions that emerged over the analysed periods. The probability that an initially un-concentrated group with *w*_*i*,*r*_ < 0.1 would establish new regional concentration over the considered period was less than 3% for USA and less than 2% for Australia. This probability sharply increases with increasing *w*_*i*,*r*_. For example, the groups with *w*_*i*,*r*_ > 0.4 had, in both countries, more than 24% probability that they would become concentrated over the analysed period (last columns of Tables 4 and 5).

**Table 4:** The average relatedness (*w*_*i*,*r*_) and regional concentrations: USA.

Upper bound of *w*_*i*,*r*_	All (2000)	Concentrated in 2000		Un-concentrated in 2000		Staying un-concentrated (2000–2010)		New concentrations (2000–2010)	
		Nm.	Of all	Nm.	Of all	Nm.	Of un-concentrated in 2000	Nm.	Of un-concentrated in 2000
0.100	105717	4978	0.047	100739	0.953	97886	0.972	2853	0.028
0.200	87904	11246	0.128	76658	0.872	71135	0.928	5523	0.072
0.300	48801	11195	0.229	37606	0.771	32562	0.866	5044	0.134
0.400	28787	9625	0.334	19162	0.666	15444	0.806	3718	0.194
0.500	17329	7792	0.450	9537	0.550	7272	0.763	2265	0.238
0.600	8030	4506	0.561	3524	0.439	2650	0.752	874	0.248
0.700	2417	1600	0.662	817	0.338	621	0.760	196	0.240
0.800	151	129	0.854	22	0.146	18	0.818	4	0.182
Total	299136	51071	0.171	248065	0.829	227588	0.917	20477	0.083

**Table 5:** The average relatedness (*w*_*i*,*r*_) and regional concentrations: Australia.

Upper bound of *w*_*i*,*r*_	All (2006)	Concentrated (2006)		Un-concentrated (2006)		Staying un-concentrated (2006–2011)		New concentrations (2006–2011)	
		Nm.	Of all	Nm.	Of all	Nm.	Of un-concentrated in 2006	Nm.	Of un-concentrated in 2006
0.100	148166	3034	0.020	145132	0.980	142807	0.984	2325	0.016
0.200	66636	7324	0.110	59312	0.890	55116	0.929	4196	0.071
0.300	52883	12254	0.232	40629	0.768	35641	0.877	4988	0.123
0.400	42508	16867	0.397	25641	0.603	21255	0.829	4386	0.171
0.500	16992	10364	0.610	6628	0.390	5026	0.758	1602	0.242
0.600	2451	1860	0.759	591	0.241	412	0.697	179	0.303
0.700	123	93	0.756	30	0.243	26	0.867	4	0.133
Total	329759	51796	0.157	277963	0.843	260283	0.936	17680	0.064

Rather counter-intuitively, Tables 4 and 5 also show that in both of the countries the probability of new immigrant concentrations slightly falls for the categories of observations with the highest density (*w*_*i*,*r*_ > 0.6). We realized that these highest density observations are typical for regions with a large number of immigrant groups already concentrated. The fact that many of foreign-born groups had already established their concentrations before *t0* naturally reduces the potential for the emergence of new concentrations in these regions between *t0* and *t1*. However, these highest density observations represent a negligible part of all observations (around 0.8% for both USA and Australia) so they are almost inconsequential with respect to the overall predictive power of *w*_*i*,*r*_.

In addition, we used a multiple regression analysis to test the predictive power of the relatedness approach when analysed together with other factors that may influence the relationship between the density and the emergence of new immigrant concentrations in regions. In the first step, we examined whether the density can be used to predict changes in the relative shares of immigrant groups in the regional population. For this purpose, we estimated the regression with a change in the relative share of an immigrant group *i* in the population of region *r* between years *t0* and *t1* (denoted as *f*_*i*,*r*,*t1-t0*_) as the dependent variable and the following independent variables:

- *w*_*i*,*r*,*t0*_ / *w*_*r*,*t0*_—relative density in *t0* as the key independent variable of interest, where *w*_*i*,*r*,*t0*_ refers to the density in *t0* and *w*_*r*,*t0*_ stands for the average density in region *r* (note that the density *w*_*i*,*r*,*t0*_ is not independent of the number of concentrated groups in particular regions so the relative density is more appropriate for the present purposes),
- *f*_*i*,*r*,*t0*_—share of a group *i* in the population of a region *r* in the initial year *t0*, required to control for initial variability in the population shares of immigrant groups in regions,
- *F*_*i*,*t1-t0*_—relative change in the total size of an immigrant group *i* over the considered period included to control for differential rates of immigration,
- *F*_*r*,*t1-t0*_—relative change in the foreign-born population in regions to control for differential regional immigration rates,

In addition, we also included a set of dummy variables to account for the macro-regional origin of individual foreign-born groups (affiliation of source countries to 13 world regions).

The results in Table 6 confirm relative density (*w*_*i*,*r*,*t0*_ / *w*_*r*,*t0*_) as a statistically significant positive predictor of changes in the regional shares of individual foreign-born groups, as expected. It holds for both USA and Australia. Note that while it was necessary to include the first two independent variables (*w*_*i*,*r*,*t0*_ */ w*_*r*,*t0*_; *f*_*i*,*r*,*t0*_;), other control variables were included purposely but were not essential to the model. However, the relationships seem to be robust across different model specifications; even when we omitted the latter group of control variables, the effects of relative density remained similarly significant.

**Table 6:** Regression results on the power of relative density in predicting the change in the regional shares of foreign-born groups.

	USA		Australia	
	B (Std. error)	Standardized B	B (Std. error)	Standardized B
(*w*_*i*,*r*,*t0*_) / (*w*_*r*,*t0*_)	2.104 (0.034)***	0.132	0.642 (0.006)***	0.216
*f*_*i*,*r*,*t0*_	-0.222 (0.001)***	-0.288	-0.193 (0.001)***	-0.394
*F*_*i*,*t1-t0*_	0.005 (0.000)***	0.039	0.001 (0.000)***	0.015
*F*_*r*,*t1-t0*_	0.000 (0.000)	0.000	0.000 (0.000)	0.000
*R* ^*2*^	0.086		0.143	
*N*	299,136		329,759	

Notes: *** statistically significant at 1%. Controlled for the macro-regional origin of foreign-born groups in terms of their affiliation to 13 world regions.

Another interesting finding which arose from these results is the significant negative effect of the initial regional population share (*f*_*i*,*r*,*t0*_), suggesting on average higher growth of foreign-born population groups in regions where they initially had lower population shares. This corresponds to the deconcentration of the foreign-born population. It can also be documented for both countries by the decreasing Gini coefficients of variation in regional foreign-born population shares (it holds both for the foreign-born population in total and for the majority—64% in USA and 85% in Australia—of individual foreign-born groups).

In the next step, we used a logistic regression with the same independent variables to examine the power of spatial relatedness measures in projecting the emergence of new concentrations of foreign-born groups as defined on the basis of *LQ*_*j*,*r*_ > 1. In this case, we only considered the set of observations pertaining to those groups that were un-concentrated in regions at *t0* (those satisfying *LQ*_*j*,*r*,*t0*_ ≤ 1). The dependent binary variable captures the emergence of new immigrant concentrations between *t0* and *t1* by attaining the value of 1 if a groups established concentration between *t0* and *t1* (meaning that *LQ*_*j*,*r*,*t1*_ > 1) and 0 otherwise. The results are presented in Table 7, which shows that for both countries the effects of relative density (*w*_*i*,*r*,*t0*_ */ w*_*r*,*t0*_) are again notable and statistically significant. This exercise thus confirms that the density can be considered as a strong predictor of the emergence of new immigrant groups in regions.

**Table 7:** Logistic regression results on the power of relative density in predicting the emergence of new concentrations of foreign-born groups.

	USA		Australia	
	B (Std. error)	Exp(B)	B (Std. error)	Exp(B)
(*w*_*i*,*r*,*t0*_) / (*w*_*r*,*t0*_)	1.555 (0,030)***	4.735	0.704 (0.012)***	2.022
*f*_*i*,*r*,*t0*_	0.083 (0.004)***	1.086	0.141 (0.006)***	1.151
*F*_*i*,*t1-t0*_	0.002 (0.000)***	1.002	0.002 (0.000)***	1.002
*F*_*r*,*t1-t0*_	0.001 (0.000)***	1.001	0.000 (0.000)	1.000
*Nagelkerke R* ^*2*^	0.045		0.063	
*N*	248,065		277,963	

Notes: *** statistically significant at 1%. Controlled for the macro-regional origin of foreign-born groups in terms of their affiliation to 13 world regions.

Finally, we analogously examined the predictive power of relative density separately for each of the 61 most populous foreign-born groups in USA (those with population size in 2010 above 100,000). For each of these groups we considered the set of un-concentrated observations in 2000 and used a logistic regression to analyse the effect of relative density on the binary dependent variable capturing the emergence of new regional concentrations in 2010. Again, we controlled for the initial regional population shares of a given group (*f*_*i*,*r*,*2000*_) as this variable can naturally be expected to affect the probability of establishing new regional concentration over subsequent period. The results appear in Table 8 and they confirmed statistically significant positive effect of relative density for the majority (79%) of the 61 foreign-born groups studied in this last exercise. In contrast, no statistically significant relationship was found in the case of 11 (18%) groups, while for another two immigrant groups (Thailand and France) the effect was statistically significant but negative.

**Table 8:** Logistic regression results on the power of relative density in predicting the emergence of new concentrations of 61 most populous foreign-born groups in USA (ordered by the population size in 2010).

	B (Std. error)		Nagelkerke R^2^	N
	(*w*_*i*,*r*,*t0*_) / (*w*_*r*,*t0*_)	*f*_*i*,*r*,*t0*_		
Mexico	1.123 (0.167)***	0.086 (0.007)***	0.164	1,838
Philippines	1.354 (0.253)***	0.267 (0.043)***	0.037	2,275
India	1.039 (0.489)**	0.381 (0.067)***	0.027	2,165
China	5.075 (0.838)***	0.408 (0.058)***	0.059	2,531
Vietnam	-0.211 (0.798)	0.219 (0.069)***	0.008	2,477
El Salvador	0.280 (0.281)	0.645 (0.088)***	0.037	2,830
South Korea	0.826 (0.256)***	0.431 (0.069)***	0.034	1,957
Cuba	1.385 (0.582)**	0.545 (0.103)***	0.029	2,849
Canada	0.434 (0.129)***	0.455 (0.080)***	0.044	1,122
Dominican Republic	2.395 (0.637)***	1.170 (0.173)***	0.081	2,969
Guatemala	-0.115 (0.273)	0.490 (0.131)***	0.009	2,608
UK	0.050 (0.136)***	0.362 (0.097)***	0.020	1,105
Jamaica	3.802 (0.620)***	1.247 (0.144)***	0.111	2,807
Germany	0.192 (0.148)	0.594 (0.123)***	0.052	692
Colombia	2.535 (0.751)***	0.825 (0.133)***	0.035	2,743
Haiti	2.610 (0.495)***	1.667 (0.273)***	0.075	2,956
Honduras	0.351 (0.324)	1.692 (0.234)***	0.034	2,579
Poland	5.111 (0.774)***	1.420 (0.143)***	0.107	2,568
Ecuador	4.016 (0.724)***	1.380 (0.376)***	0.049	2,921
Peru	0.384 (0.782)	1.516 (0.255)***	0.024	2,754
Russia	2.462 (0.556)***	1.875 (0.174)***	0.080	2,405
Italy	2.505 (0.663)***	1.374 (0.147)***	0.080	2,439
Taiwan	4.315 (1.114)***	0.930 (0.248)***	0.030	2,654
East Europe, nfd	2.318 (0.577)***	2.523 (0.242)***	0.083	2,365
Japan	-0.191 (0.230)	0.928 (0.182)***	0.030	1,896
Brazil	1,704 (1.155)	2.469 (0.415)***	0.043	2,590
Iran	2.983 (1.105)***	1.143 (0.350)***	0.025	2,770
Ukraine	8.045 (1.200)***	1.775 (0.283)***	0.103	2,741
Pakistan	9.975 (1.615)***	2.319 (0.355)***	0.073	2,598
East Africa, nfd	5.431 (1.378)***	2.513 (0.558)***	0.031	2,519
Guyana	4.108 (0.632)***	4.749 (0.474)***	0.170	2,902
Nicaragua	1.138 (0.566)**	2.066 (0.461)***	0.021	2,865
Trinidad and Tobago	4.156 (0.694)***	3.803 (0.425)***	0.113	2,777
Caribbean, nfd	5.613 (0.775)***	4.133 (0.467)***	0.113	2,717
Hong Kong	4.795 (1.161)***	1.827 (0.482)***	0.047	2,839
Thailand	-1.395 (0.428)***	2.218 (0.403)***	0.048	2,226
Nigeria	5.284 (0.836)***	4.101 (0.692)***	0.092	2,739
Laos	-1.236 (0.764)	2.602 (0.450)***	0.031	2,574
Portugal	3.755 (1.001)***	4.011 (0.496)***	0.084	2,924
West Africa, nfd	5.053 (0.680)***	5.999 (0.998)***	0.109	2,833
Argentina	10.475 (1.490)***	4.343 (0.620)***	0.109	2,700
Venezuela	7.388 (1.184)***	4.301 (0.746)***	0.074	2,690
Romania	6.295 (1.034)***	4.339 (0.531)***	0.073	2,532
France	-0.854 (0.319)***	2.675 (0.450)***	0.056	1,964
Bangladesh	5.899 (0.772)***	5.477 (1.174)***	0.121	2,870
Cambodia	3.212 (0.922)***	3.755 (0.716)***	0.043	2,790
West Asia, nfd	8.039 (1.061)***	4.163 (0.728)***	0.103	2,659
Ethiopia	3.630 (0.543)***	2.980 (2.002)	0.049	2,889
Greece	7.204 (1.339)***	3.788 (0.514)***	0.078	2,576
Israel	4.341 (0.895)***	4.948 (0.893)***	0.086	2,851
Egypt	9.497 (1.313)***	3.841 (0.775)***	0.122	2,701
Ireland	0.831 (0.621)	3.745 (0.445)***	0.053	2,393
North Africa and the Middle East, nfd	9.072 (1.383)***	5.917 (1.060)***	0.093	2,637
Africa, n.e.c.	6.354 (0.754)***	9.702 (1.538)***	0.116	2,753
Lebanon	6.892(1.057)***	4.365 (0.864)***	0.107	2,764
Iraq	5.551 (0.970)***	5.382 (1.539)***	0.083	2,923
Bosnia and Hercegovina	6.068 (0.876)***	0.753 (1.845)	0.061	2,846
Oceania, n.e.c.	-0.453 (0.777)	7.535 (1.179)***	0.030	2,762
Ghana	5.724 (0.668)***	8.933 (1.735)***	0.137	2,853
Panama	-3.575 (0.723)***	3.384 (0.756)***	0.063	2,359
Serbia and Montenegro	4.993 (0.980)***	4.784 (0.720)***	0.060	2,586

Notes:

*** significant at 1%

** significant at 5%. N refers to the number of un-concentrated *D*_*i*,*j*_ observations in 2000.

The finding that the predictive power of relative density varies across particular foreign-born groups should not come as a surprise. In fact, many of the immigrant groups with insignificant or negative beta coefficients obtained for the relative density variable in Table 8 are known for their distinct spatial behaviour that makes our spatial relatedness approach less applicable. For example, the most populous of these groups in terms of Vietnamese immigrants has a history of refugees participating in governmental settlement programs that has specifically influenced their spatial distribution. Similarly, large inflows of migrants from the three Central American countries with the insignificant relative density coefficients: El Salvador, Guatemala, and Honduras originated in civil wars in these countries. Together with Panama (as a foreign-born group whose population in USA decreased over the analysed period), these three Central American groups appeared unrelated to other immigrant groups shown in the network visualisation in S1 Fig. The latter also holds for the foreign-born groups from Thailand, Laos, or Oceania n.e.c. Another reason for the insignificant coefficients revealed for some of the immigrant groups can also be explained by their stable (France, Japan) or even decreasing (Laos, Ireland, Germany) population size in USA. These and a few other exceptions apart, the analysis at the level of individual immigrant groups also corroborated that the density variables often contain interesting information that can be helpful in making predictions about the future composition of regional populations.

### Examples of application

**Fig 4:**
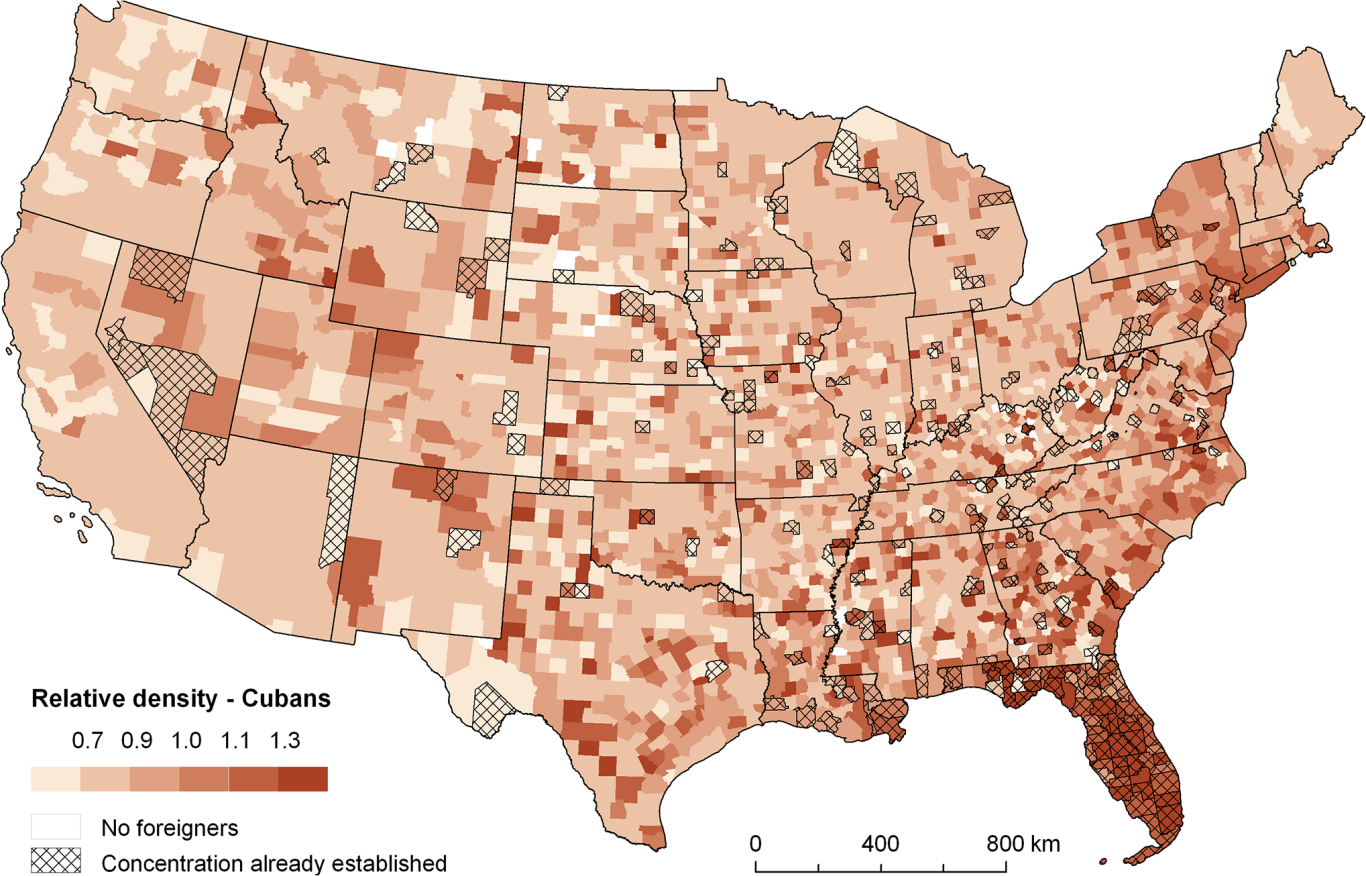
Relative group densities for Cubans (2010). Note: relative density (*w*_*i*,*r*_ / *w*_*r*_) mirrors the probability of establishing new regional concentration.

**Fig 5:**
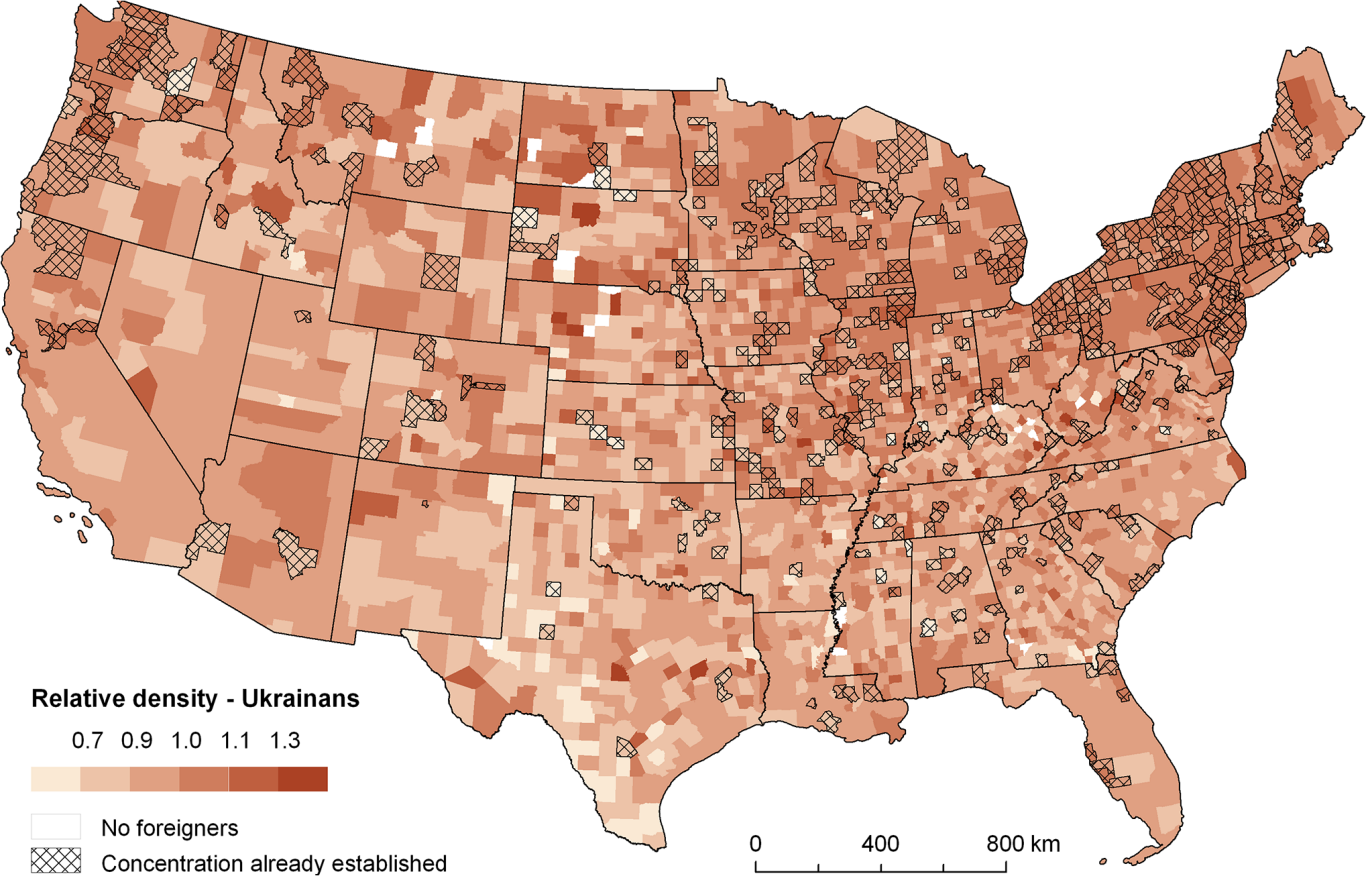
Relative group densities for Ukrainians (2010). Note: relative density (*w*_*i*,*r*_ / *w*_*r*_) mirrors the probability of establishing new regional concentration.

**Fig 6:**
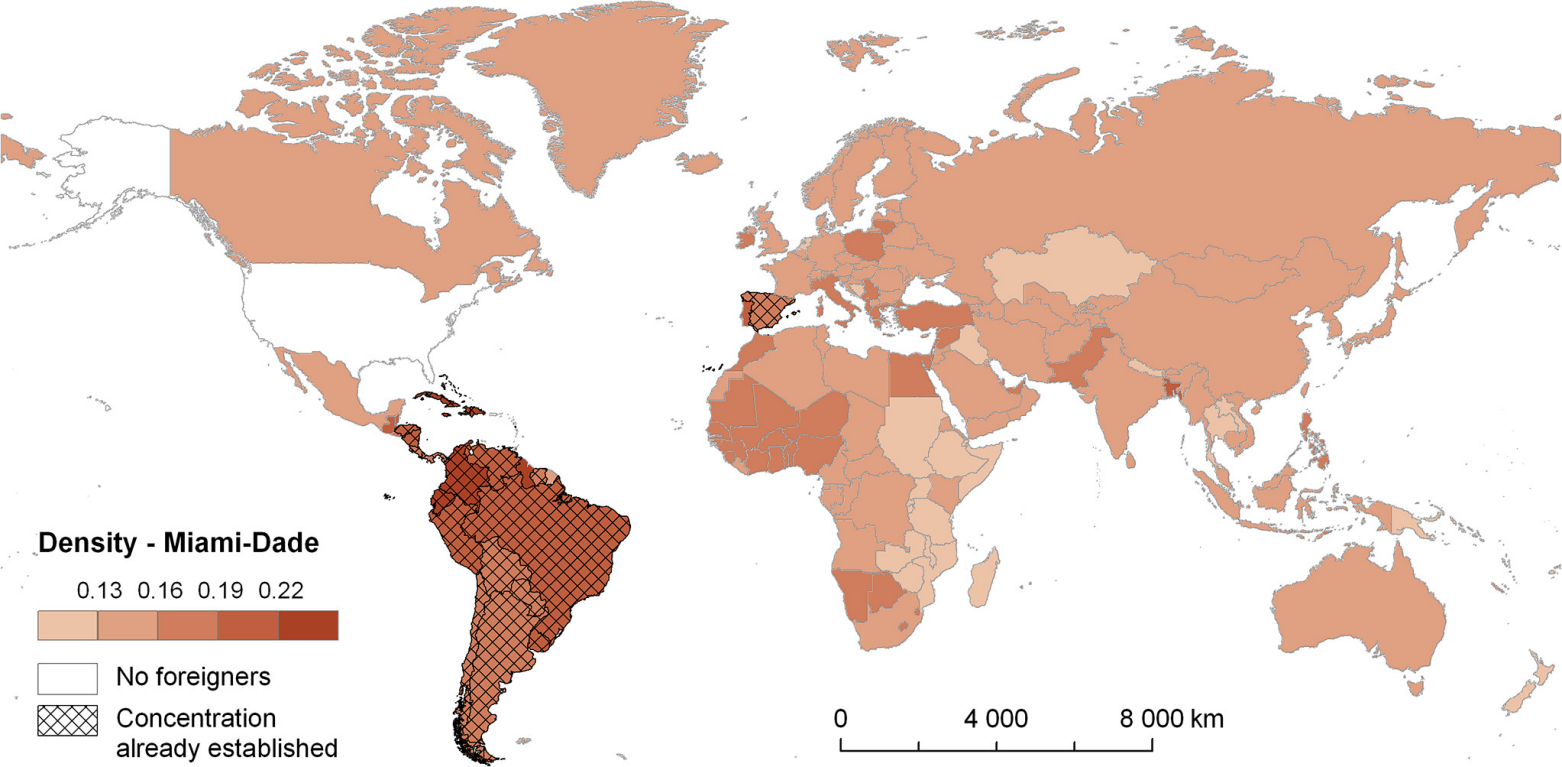
Density levels (*w*_*i*,*r*_) for particular foreign-born groups and Miami-Dade county (2010). Note: The darker colours, the higher probability of establishing concentration in Miami-Dade.

**Fig 7:**
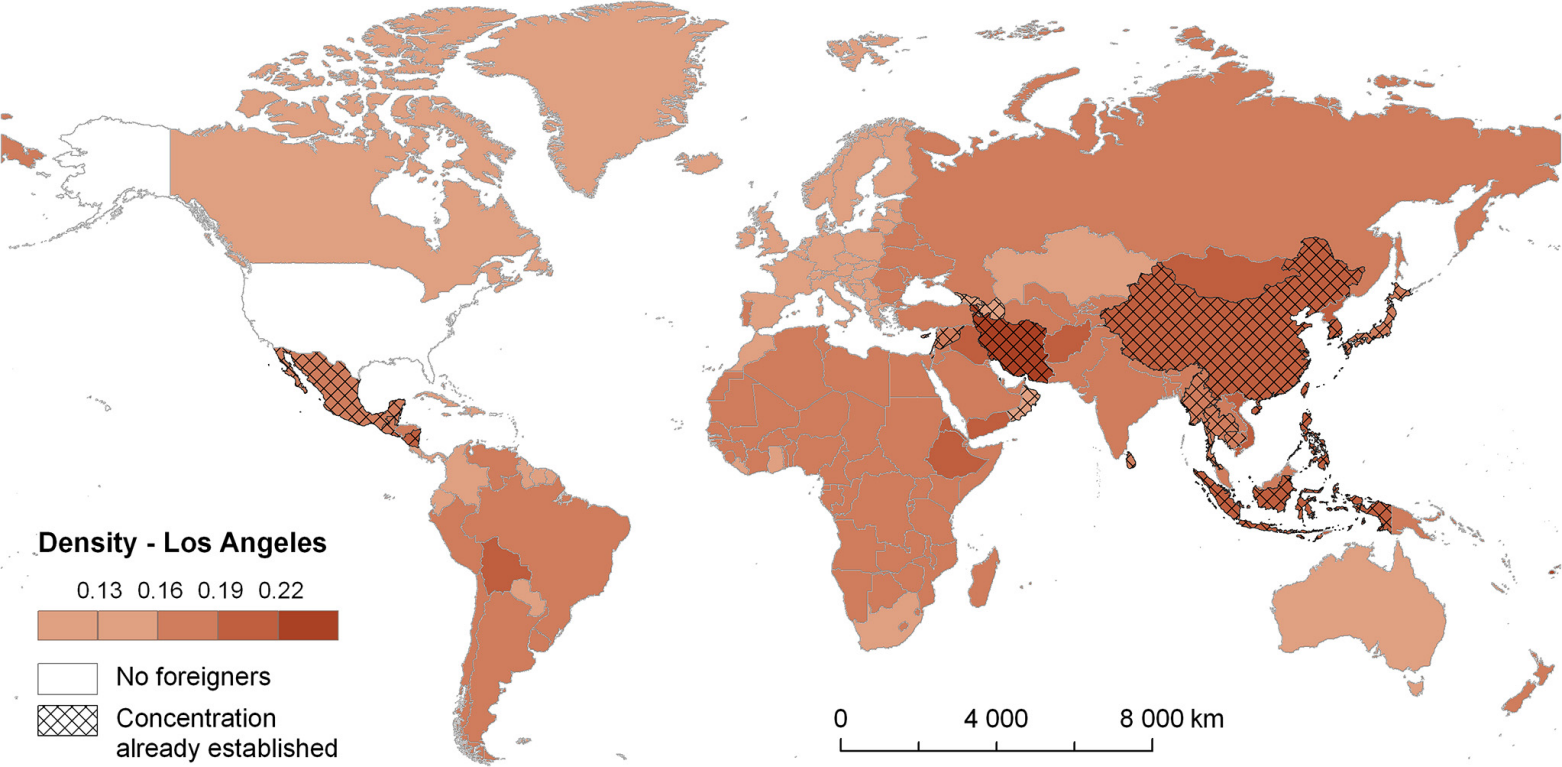
Density levels (*w*_*i*,*r*_) for source countries of foreign-born groups and Los Angeles (2010) Note: The darker colours, the higher probability of establishing concentration in Los Angeles.

Finally, Figs 4–7 present some examples showing a practical application of the spatial relatedness approach. The maps in Figs 4 and 5 depict the relative density (*w*_*i*,*r*_ / *w*_*r*_) for two selected foreign-born groups, namely Cubans and Ukrainians, computed from the most recent US 2010 data. Darker colours mean higher relative density, that is, higher spatial relatedness to the set groups concentrated in a region. In addition, a cross-hatched pattern signifies regions where the given group has already established a concentration. As is well known, Cubans are mostly spatially concentrated in Florida. This is also signified by the cross-hatched pattern of Florida counties in Fig 4, while they dark colours suggest that these Cuban concentrations are unlikely to disappear. The emergence of new concentrations of Cubans can nevertheless be expected in a number of counties along the Atlantic coast up to the New York state. By contrast, there is a number of other counties more on the west marked by a cross-hatched pattern but light colours in Fig 4, where Cubans have already been concentrated but have a comparatively higher probability of de-concentration in the future. In addition, Fig 5 shows a north-south gradient recognizable with respect the spatial concentrations of Ukrainians. It also suggests that this pattern is likely to deepen in the future.

For two selected examples, namely Miami-Dade and Los Angeles counties, Figs 6 and 7 depict the density (*w*_*i*,*r*_) for migrant groups from individual source countries across the world. The density corresponds to the spatial relatedness between immigrant groups from the respective countries and the pool of other groups already concentrated in Miami-Dade (Fig 6) and Los Angeles (Fig 7). As such, the darker is a country colour the higher is the probability that immigrants born in this country will establish concentration in these counties. Although both of them represent primary examples of multi-ethnic counties, the patterns revealed in Figs 6 and 7 differ considerably. Fig 6 reflects major role of known concentrations of Latino and Hispanic groups in Miami associated with a comparatively high probability of establishing new concentration identified for immigrants born especially in several Mediterranean countries. Finally, Fig 7 reflects even more diverse population composition of Los Angeles with the prominence of concentrations (i.e. *LQ*_*i*,*r*_ > 1) of immigrants from Mexico and several West Asian and East and South East Asian countries. This heterogeneous composition of concentrated groups determines relatively high density indicated for the most of Asian, African, and Latin American immigrant groups.

## Conclusions

Temporal trends (relatedness in time) and spatial patterns (relatedness in space) can be regarded as two similarly essential sources of inference for population forecasting. While the former has attracted a lot of attention (extrapolations in time have been the most traditional method of making population projections), we have attempted to show that information derived from the spatial patterns of population distribution has yet to be fully utilized. In this paper we applied a so-called spatial relatedness approach to examine the process of regional population diversification and the emergence of new immigrant concentrations at the regional level in USA and Australia. The basic assumption behind this approach is that the spatial proximity between foreign-born groups mirrors, at least to a certain extent, various other aspects of their relatedness. Given this, the central goal of the paper was to show that the spatial relatedness, as determined by an examination of joint concentrations of foreign-born groups in regions, can provide a useful instrument for the analysis and projection of regional population change.

Because of its high complexity and large number of push and pull factors behind both international and intra-national migration choices, the geography of immigration necessarily represents an emergent socio-spatial system with limited predictability [7, 8, 47]. Although necessarily uncertain, the projections of regional immigration have been of high value in determining policy, especially in countries where immigration drives regional population dynamics. An intuitive, and perhaps the most common, method of projecting regional immigration is to consider total national inflows (or their estimates) and allocate immigration proportionally to the stocks of foreigners already settled in regions, possibly also accounting for variation in recent inflows and for various demographic or socioeconomic characteristics of regions that are expected to influence immigration [4]. However, this kind of temporal multiregional extrapolation based on a simple reflection of the migration social networks hypothesis, can be imperfect in many situations. This may, for example, be the case during turbulent changes in the volume and structure immigration associated with inflows of new immigrant groups and emergence of their new spatial concentrations [48]. The US and Australian data examined here provide examples of such situations, as suggested above (and also documented by the negative beta coefficients of the stocks variable (*f*_*i*,*r*,*t0*_) in Table 6 related to the process of spatial dispersion of foreigners).

The main contribution of this study is the proposition of a new component, namely the spatial relatedness term, which can add a precision to regional immigration estimates disaggregated by foreign-population groups. As in traditional approaches, we assumed that the current composition of immigrant groups concentrated in a region affects its future state. However, instead of considering the temporal aspect by extrapolating the regional population composition over time, the component proposed here makes use of the information captured by spatial relatedness measures to estimate the direction of future population diversification and the potential of immigration disaggregated by population subgroups and regions.

Obviously, this technique can analogously be used for other population data disaggregated by spatially defined subpopulations and combined with other techniques of multiregional population modelling [9]. In addition, the analysis of spatial relatedness is scalable and it is similarly applicable to any other definition of regions. For example, similar analysis of spatial relatedness could be done with the data on the spatial distribution of immigrants within major cities or metropolitan areas. This could provide valuable practical insight on the relationships between particular immigrant groups in a given local context. Note that an analysis based on national data (as in this paper) makes an implicit assumption about the spatial homogeneity of the processes operating behind the relatedness figures. Therefore, the comparison of the structure of spatial relatedness links based on data for different spatial systems can be another interesting exercise. Already the brief comparison of the US and Australian figures above suggests some apparent parallels in these two sets of results, mostly reflecting the geographical and cultural proximity of foreign-born groups.

## Supporting Information

S1 Fig The patterns of spatial relatedness between foreign-born groups and native population in USA. (TIFF)

S2 Fig The patterns of spatial relatedness between foreign-born groups and native population in Australia. (TIFF)
