# Supplementary figures and images for: The Emergence of Regional Immigrant Concentrations in USA and Australia: A Spatial Relatedness Approach
Source: PLoS One. 2015 May 12;10(5):e0126793. doi: 10.1371/journal.pone.0126793 (PMC4429065; doi:10.1371/journal.pone.0126793)

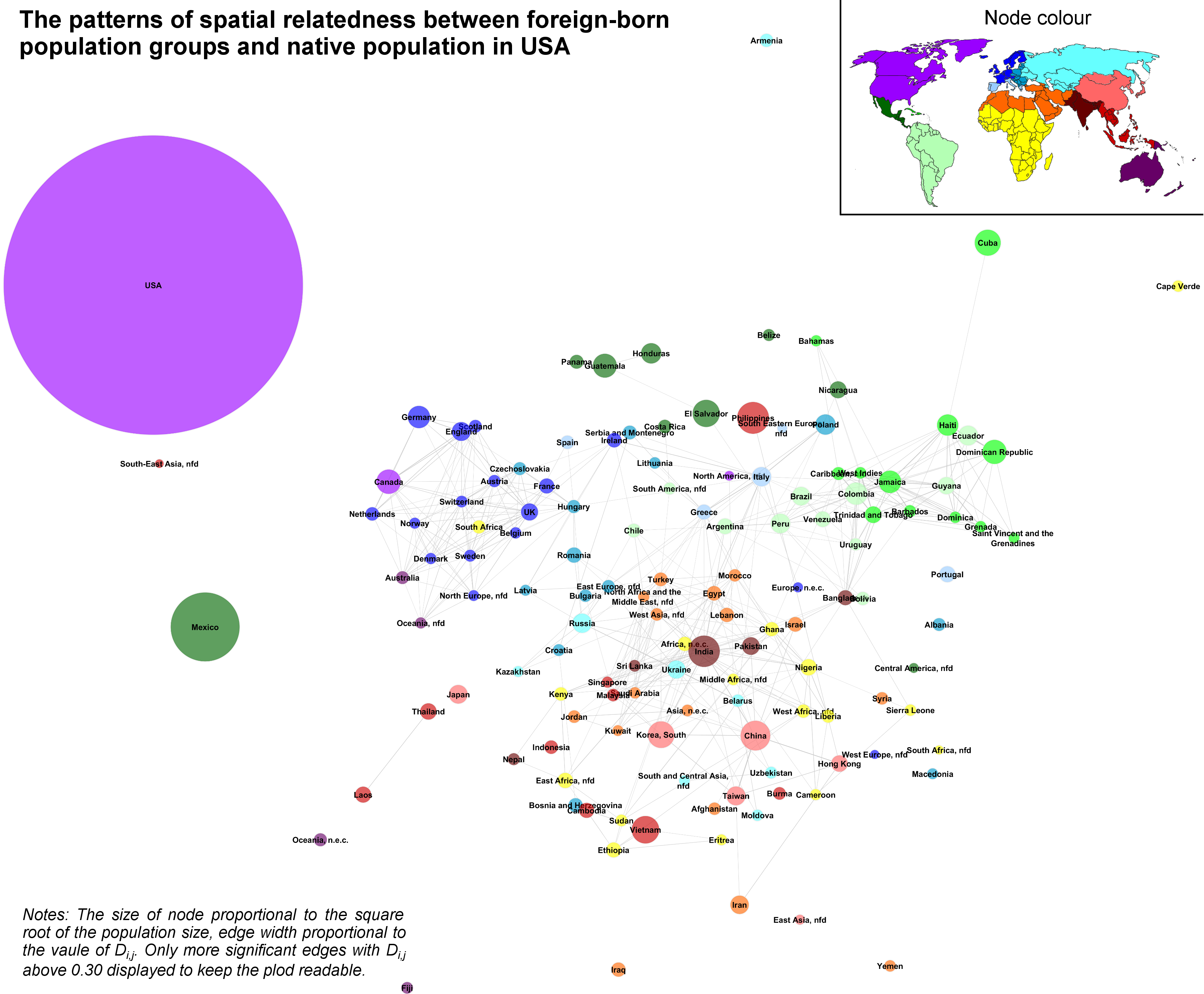

Supplement: S1 Fig — (TIFF) [file pone.0126793.s001.tiff]

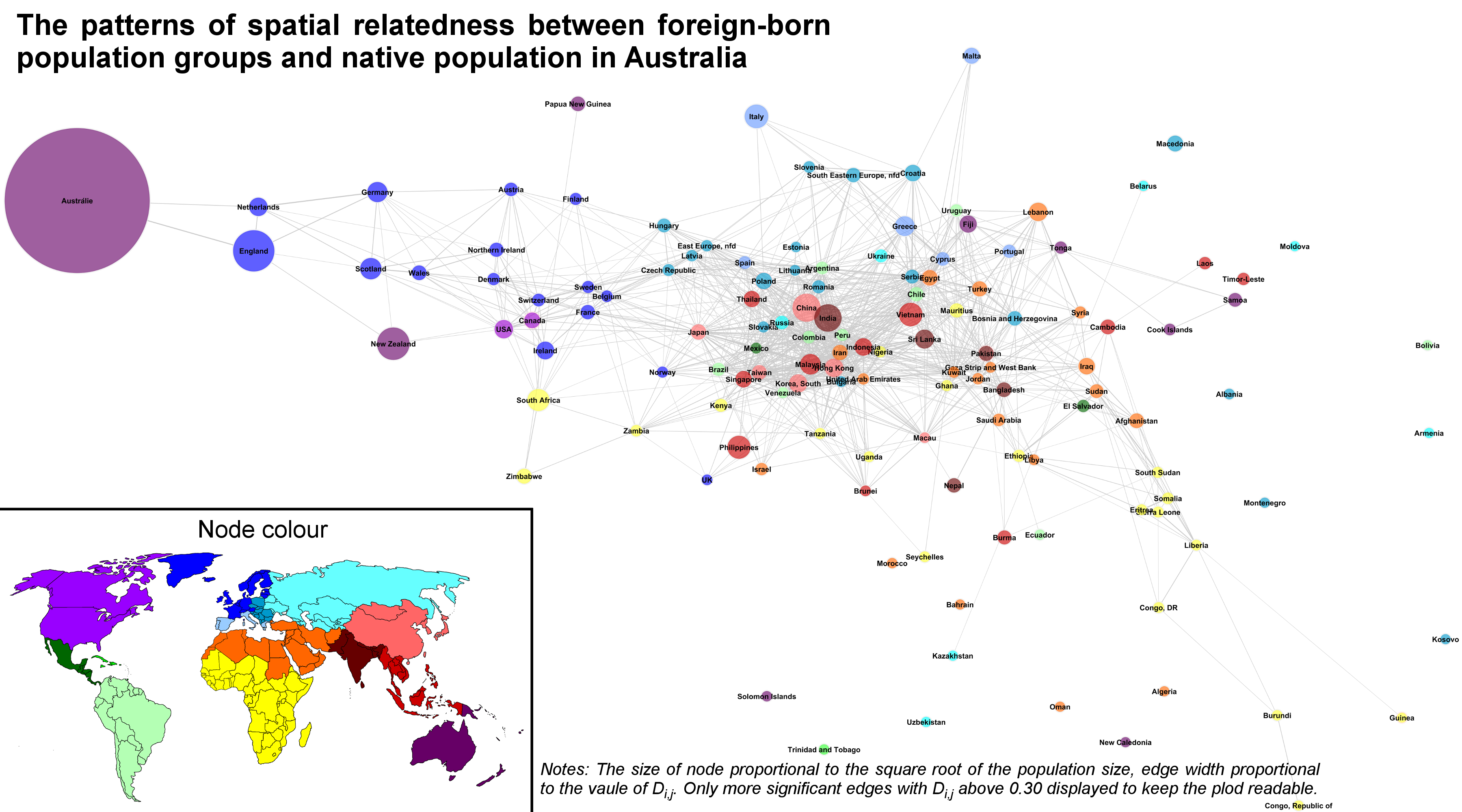

Supplement: S2 Fig — (TIFF) [file pone.0126793.s002.tiff]
